# Gene Mutation Spectrum among Alpha-Thalassaemia Patients in Northeast Peninsular Malaysia

**DOI:** 10.3390/diagnostics13050894

**Published:** 2023-02-27

**Authors:** Divashini Vijian, Wan Suriana Wan Ab Rahman, Kannan Thirumulu Ponnuraj, Zefarina Zulkafli, Rosnah Bahar, Norafiza Yasin, Syahzuwan Hassan, Ezalia Esa

**Affiliations:** 1 School of Dental Sciences, Universiti Sains Malaysia, Kubang Kerian 16150, Kelantan, Malaysia; 2 Hospital Universiti Sains Malaysia, Kubang Kerian 16150, Kelantan, Malaysia; 3 Department of Hematology, School of Medical Sciences, Universiti Sains Malaysia, Kubang Kerian 16150, Kelantan, Malaysia; 4 Haematology Unit, Cancer Research Centre, Institute for Medical Research, Shah Alam 40170, Selangor, Malaysia

**Keywords:** α-thalassaemia, genotype, mutation, globin, prevalence, molecular analysis

## Abstract

(1) Background: Alpha (α)-thalassaemia is a genetic disorder that affects 5% of the world population. Deletional or nondeletional mutations of one or both *HBA1* and *HBA2* on chromosome 16 will result in reduced production of α-globin chains, a component of haemoglobin (Hb) that is required for the formation of red blood cells (RBCs). This study aimed to determine the prevalence, haematological and molecular characterisations of α-thalassaemia. (2) Method: The parameters were based on full blood count, high-performance liquid chromatography and capillary electrophoresis. The molecular analysis involved gap-polymerase chain reaction (PCR), multiplex amplification refractory mutation system-PCR, multiplex ligation-dependent probe amplification and Sanger sequencing. (3) Results: With a total cohort of 131 patients, the prevalence of α-thalassaemia was 48.9%, leaving the remaining 51.1% with potentially undetected α gene mutations. The following genotypes were detected: -α^3.7^/αα (15.4%), -α^4.2^/αα (3.7%), --^SEA^/αα (7.4%), α^CS^α/αα (10.3%), α^Adana^α/αα (0.7%), α^Quong Szeα^/αα (1.5%), -α^3.7^/-α^3.7^ (0.7%), α^CS^α/α^CS^α (0.7%), -α^4.2^/α^CS^α (0.7%), –^SEA^/α^CS^α (1.5%), –^SEA^/α^Quong Sze^α (0.7%), -α^3.7^/α^Adana^α (0.7%), --^SEA^/-α^3.7^ (2.2%) and α^CS^α/α^Adana^α (0.7%). Indicators such as Hb (*p* = 0.022), mean corpuscular volume (*p* = 0.009), mean corpuscular haemoglobin (*p* = 0.017), RBC (*p* = 0.038) and haematocrit (*p* = 0.058) showed significant changes among patients with deletional mutations, but not between patients with nondeletional mutations. (4) Conclusions: A wide range of haematological parameters was observed among patients, including those with the same genotype. Thus, a combination of molecular technologies and haematological parameters is necessary for the accurate detection of α-globin chain mutations.

## 1. Introduction

Haemoglobinopathies are inherited disorders caused by alterations in the globin genes (α and β), thus affecting the production of their proteins and synthesis of haemoglobin (Hb) in red blood cells (RBCs). The mutated globin genes may produce abnormal proteins that determine the Hb variant, or it may cause a reduction in the affected globin expression that subsequently leads to alpha (α-) or beta (β-) thalassaemia [1]. The World Health Organization (WHO) estimates that 5% of the world’s population are carriers of α-thalassaemia, with the majority being Southeast Asians [1]. Besides Southeast Asia, the disorder is also prevalent in Mediterranean countries, the Middle East, Central Asia, India, Southern China, North Africa, and South America. The mutated globin genes have different combinations that can produce more than 60 thalassaemia syndromes, making Southeast Asia the region with the most complex disease genotype [2].

Alpha (α)- and beta (β)-thalassaemia are caused by defects in *HBA* and *HBB* genes, respectively. Mutations of *HBA1* and *HBA2* on chromosome 16 will result in α-thalassaemia. A meta-analysis reported the prevalence of α-thalassemia in 22.5% of the population in Southeast Asia and 17.5% in Malaysia alone [3]. Individuals with a single α-globin gene defect are silent carriers who will have no manifestation of the disease. Individuals who inherit two defective α-globin chains are known as α-thalassaemia traits and may display mild symptoms of anaemia. When three α-globin chains are affected, it results in Hb H, which is a moderate to severe form of the disease that requires lifelong health monitoring [4]. The most severe form of α-thalassaemia is Hb Barts, in which all four α-globin genes are defective, and it is not compatible with life [4]. This condition mostly affects foetuses, which may develop hydrops fetalis and end up stillborn.

α-thalassaemia may occur due to deletional or nondeletional mutations in the α-globin gene, and the former has been observed to be more common. However, the clinical outcome among nondeletional α-thalassaemia individuals is more severe compared with deletional individuals. This is because nondeletional mutations usually involve *HBA2*, which has higher expression than *HBA1* in a 3:1 ratio [5]. Thus, it has a huge impact on the production of α-globin chains. The most common single deletional mutations detected in Southeast Asia are -α^3.7^ and -α^4.2^. Apart from those, the most common double-gene deletions reported worldwide are -(α)^20.5^, --^SEA^, --^MED^, --^THAI^ and --^FIL^ [6]. The common nondeletional variants of α-thalassaemia mutations reported in Southeast Asia are Hb Constant Spring (CS), Hb Quong Sze and Hb Adana, whereas codon 30 and codon 35 mutations are rarely identified [7].

Therefore, the aim of this study was to investigate the haematological and molecular characterisations of α-globin gene mutations and their variants in patients diagnosed with α-thalassaemia at the Hospital Universiti Sains Malaysia. The institution is an established teaching hospital of a public university in the northeast state of Kelantan in Peninsular Malaysia. It mostly serves as a referral centre for medical treatments on the east coast of the peninsula.

## 2. Materials and Methods

### 2.1. Patient Screening and Selection

This cross-sectional study involved 131 patients suspected of having α-thalassaemia based on haematological parameters (mean corpuscular haemoglobin (MCH) < 27 pg and mean corpuscular volume (MCV) < 80 fl). Those with normocytic and normochromic indices were excluded. Upon obtaining informed consent, two millilitres of peripheral blood samples were collected in ethylenediaminetetraacetic acid (EDTA) tube. The Hb concentrations and red cell indices were determined using an automated blood cell counter. The quantitation of Hb was performed using an automated high performance liquid chromatography (HPLC) and capillary electrophoresis (CE) system. Molecular tests were conducted on patients suspected of α-thalassaemia. This study was approved by the Medical Research and Ethics Committee (NMMR-21-606-58737) and the Human Research Ethics Committee of Universiti Sains Malaysia (USM/JEPeM/20020104).

### 2.2. Haematological Analysis

The Sysmex XN-1000 automated haematology analyser (Sysmex America Inc., Lincolnshire, IL, USA) was used to obtain the parameters of whole blood samples (MCH, MCV and MCHC). Preparation of blood samples for HPLC and CE was carried out according to the institution’s routine protocols. In HPLC, the VARIANT II β-Thalassemia Short Programme (Bio-Rad Inc., Hercules, CA, USA) was used to detect the level of Hb A_2_ and Hb F. The programme utilised the cation exchange principle to separate Hb variants in patients’ blood. The presence of variants such as Hb H, Hb Barts and Hb Constant Spring was quantified through CE using the CAPILLARYS 2 Flex Piercing instrument (SEBIA, Lisses, France).

### 2.3. DNA Extraction

DNA was extracted from patients’ whole blood using the QIAamp DNA Blood Minikit (Qiagen GmBH, Dusseldorf, Germany) according to the manufacturer’s instructions. The concentration and purity of extracted DNA were determined using the Eppendorf BioPhotometer spectrophotometer (Eppendorf GmBH, Hamburg, Germany).

### 2.4. Multiplex Gap-PCR

The extracted DNA was subjected to multiplex gap-polymerase chain reaction (gap-PCR) to detect deletions in α-globin genes. The multiplex gap-PCR contained mutation-specific primers for amplification of --^SEA^, -α^3.7^, -α^4.2^, --^FIL^, --^THAI^, -(α)^20.5^ and --^MED^. All positive controls for known genotypes were provided by the Haematology Unit of the Cancer Research Centre at the Institute for Medical Research in Selangor, Malaysia. The primer sequences shown in Table S1 are available in the “Supplementary Materials” and were based on a previous study [8]. Amplification was carried out in a 25 µL reaction volume consisting of 50 ng of DNA template, 2.5 U of HotStarTaq master mix (Qiagen, Dusseldorf, Germany), 1× Q solution (Qiagen, Dusseldorf, Germany), forward and reverse primers and nuclease-free water. The concentration of each primer used is shown in Table S1. PCR was carried out in a thermocycler (Bio-Rad, Hercules, CA, USA), beginning with initial denaturation at 96 °C for 15 min, followed by 30 cycles of denaturation (98 °C, 45 s), annealing (64 °C, 1 min 30 s) and extension (72 °C, 2 min 30 s). In the final extension, the reaction was allowed to run at 72 °C for 5 min before terminating at 8 °C.

### 2.5. Multiplex Amplification Refractory Mutation System–Polymerase Chain Reaction

Multiplex amplification refractory mutation system–polymerase chain reaction (ARMS-PCR) was performed to detect nondeletional mutations in the α-globin gene. The method can detect common mutations that occur in the initiation codon (c.2delT), codon 30 (c.91_93delGAG), codon 35 (c.106T>C), Hb Adana (c.179G>A), Hb Quong Sze (c.377T>C) and Hb Constant Spring (c.427T>C). The primer sequences used had been stated in a previous study [7]. Amplification was carried out in a 25 µL reaction volume consisting of 50 ng of DNA template, 2.5 U of HotstarTaq master mix (Qiagen, Dusseldorf, Germany), 1× Q solution (Qiagen, Dusseldorf, Germany), forward and reverse primers and nuclease-free water. The concentration of each primer used shown in Table S2 is available in the “Supplementary Materials”. PCR began with an initial denaturation at 96 °C for 15 min, followed by 30 cycles of denaturation (98 °C, 45 s), annealing (62.4 °C, 1 min) and extension (72 °C, 2 min 30 s). Final extension was performed at 72 °C for 5 min before the PCR products were subsequently cooled to 8 °C.

### 2.6. Duplex-PCR

Samples with mutations identified through multiplex ARMS-PCR were further tested with duplex-PCR to determine zygosity for the detected mutation (zygosity test). The zygosity test helps to identify whether the patient carries homozygous or heterozygous nondeletional mutations.

Each designed PCR reaction volume consisted of a 25 µL reaction volume of 2.5 U HotstarTaq master mix, 1× Q solution, forward and reverse primers and nuclease-free water. The designed master mix was only able to detect the heterozygosity for each mutation. PCR was carried out under the following conditions: initial denaturation at 96 °C for 15 min, followed by 30 cycles of denaturation at 98 °C for 45 s, annealing at 62.4 °C for 1 min, extension at 72 °C for 2 min 15 s and final extension at 72 °C for 5 min.

### 2.7. Agarose Gel Electrophoresis

The PCR products of multiplex gap-PCR were subjected to electrophoresis at 110 V for 40 min in a 1% agarose gel, whereas the products of multiplex ARMS- and duplex-PCR were run on a 2% agarose gel. All gels were stained with Florosafe DNA stain (1st BASE, Singapore) and visualised under ultraviolet (UV) rays in an image analyser.

### 2.8. Multiplex Ligation-Dependent Probe Amplification

The DNA samples of subjects who were determined to have no mutations in their α-globin genes after multiplex gap-PCR and ARMS-PCR were subjected to multiplex ligation-dependent probe amplification (MLPA) to screen for rare deletions. The SALSA MLPA Probemix P140-C1 HBA (MRC Holland, Amsterdam, The Netherlands) was used according to the manufacturer’s instructions. The SALSA MLPA reagent kit contained 45 MLPA probes that could be used to detect sequences in the α-globin gene cluster and their flanking regions. A comparative analysis of fragments was performed using the probe manufacturer’s Coffalyser.Net software to identify the deleted regions in *HBA*.

### 2.9. Sanger Sequencing

All the samples that were negative for mutations in multiplex gap-PCR, multiplex ARMS-PCR and MLPA were subjected to Sanger sequencing for detection of rare nondeletional mutations. The target genes of *HBA1* and *HBA2* were amplified separately before being sequenced. The master mix consisted of 50 µL reaction volume, 50 ng of DNA template, 2.5 U HotstarTaq plus master mix (Qiagen, Dusseldorf, Germany,) 0.5× Q solution (Qiagen, Dusseldorf, Germany), forward and reverse primers and nuclease-free water. The PCR conditions for the amplification of target genes used were an initial denaturation at 96 °C for 5 min, followed by 35 cycles of denaturation at 98 °C for 45 s, annealing at 64 °C for 1 min 30 s and extension at 72 °C for 2 min 30 s. The amplified target genes were sent to Apical Scientific Sdn Bhd and Institute for Research in Molecular Medicine, Universiti Sains Malaysia, for sequencing. The results were analysed using BioEdit version 7.2. The wild-type sequences of target genes were retrieved from the National Center for Biotechnology Information (NCBI) under accession number NG 000006.1 and aligned with the sequencing results for mutation identification.

### 2.10. Statistical Analysis

All the data were analysed using IBM SPSS version 22 (IBM Corp, Armonk, NY, USA). The Kruskal–Wallis test with Dunn–Bonferroni post hoc correction was used to determine whether there were significant variations between haematological parameters according to individual α-thalassaemia mutations. A *p*-value of <0.05 was considered significant.

## 3. Results

Among 131 subjects, 72 (55.0%) were female, and the remaining 59 (45.0%) were male. Most of them were aged 21 and above (*n* = 79, 60.3%), followed by 1–12-year-olds (*n* = 40, 30.5%), 13–20-year-olds (*n* = 10, 7.6%) and 1–11-month olds (*n* = 2, 1.5%). Ethnically, 96.2% of the subjects were Malays, followed by 3.1% Chinese and 0.8% Indians.

**Figure 1:**
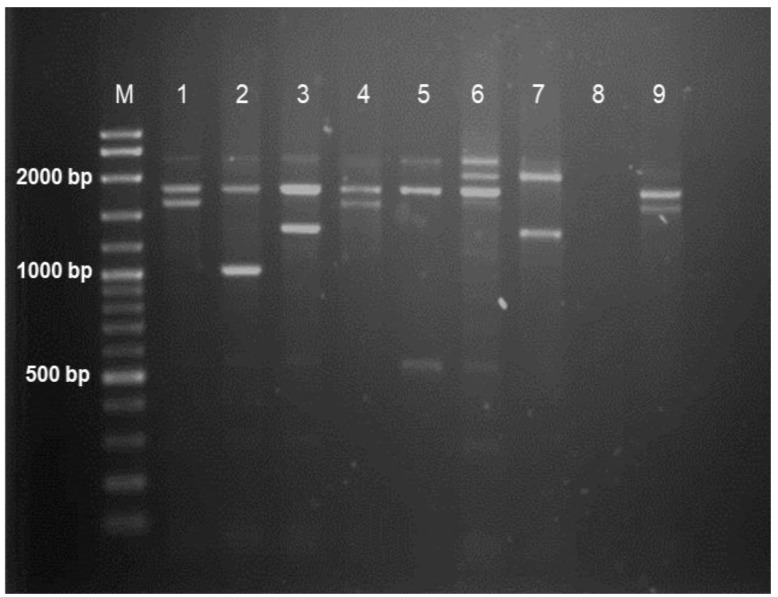
Representative gel image for α deletional mutations: Lane M: 100 bp plus ladder; Lane 1: heterozygous 4.2 mutation (-α^4.2^/αα); Lane 2: heterozygous 20.5 mutation (-α^20.5^/αα); Lane 3: heterozygous SEA mutation (--^SEA^/αα); Lane 4: heterozygous 4.2 mutation (-α^4.2^/αα); Lane 5: heterozygous Fil mutation (--^FIL^/αα); Lane 6: heterozygous 3.7 mutation (-α^3.7^/αα); Lane 7: compound heterozygous (--^SEA^/-α^3.7^); Lane 8: negative control; Lane 9: heterozygous 4.2 mutation (-α^4.2^/αα).

**Figure 2:**
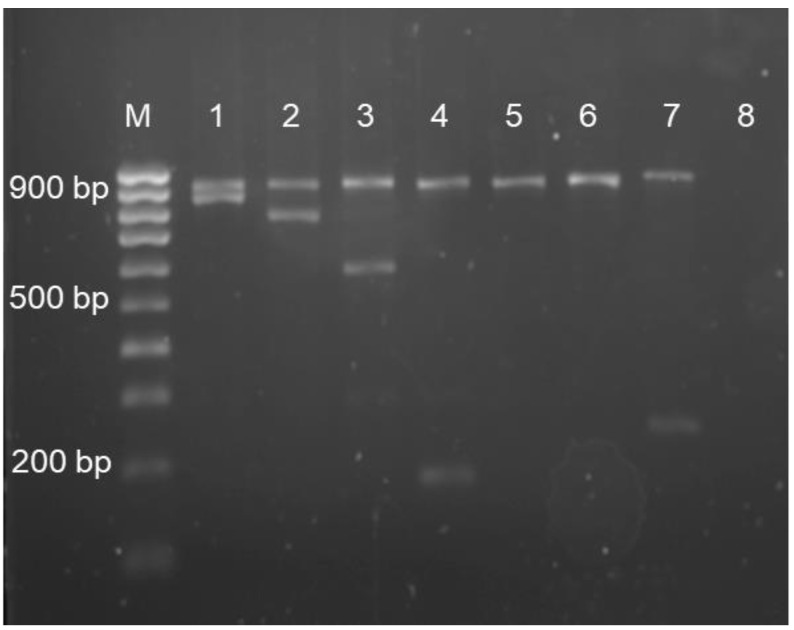
Representative gel image for α nondeletional mutations: Lane M: 100 bp ladder; Lane 1: initiation codon mutation; Lane 2: codon 30 mutation; Lane 3: codon 59 mutation (Hb Adana); Lane 4: codon 142 mutation (Hb Constant Spring); Lane 5–6: normal α; Lane 7: codon 142 mutation (Hb Constant Spring); Lane 8: negative control.

Multiplex gap-PCR and ARMS-PCR determined that 64 (48.9%) subjects had α-thalassaemia. Among the α-thalassaemia cases identified, 37 (57.8%) carried deletional mutations in *HBA*, whereas 18 (28.1%) had nondeletional mutations. The remaining nine subjects (14.1%) were identified as having compound heterozygous for α-thalassaemia. However, there were no mutations detected in the MLPA and Sanger sequencing analyses, leaving 67 (51.1%) subjects with no detectable mutations in *HBA1* and *HBA2*. There were single (-α^3.7^/αα and -α^4.2^/αα) and double-gene (--^SEA^/αα) deletional mutations detected. The point mutations identified were as follows: α^CS^α/αα, α^Adana^α/αα and α^Quong Sze^α/αα. The type of α-globin gene mutations is listed in Table 1. Figure 1 and Figure 2 show the PCR genotype results for common deletional and nondeletional α-globin gene mutations, respectively.

### Analyses of Haematological Parameters

The haematological parameters, including Hb, MCV, MCH, MCHC, red blood cells (RBCs), red cell distribution width (RDW), haematocrit (Hct) and quantification of Hb A_2_, Hb A and Hb F were analysed for differences among genotypes. As shown in Table 2, the Kruskal–Wallis test found significant differences between deletional mutations in Hb, MCV, MCH, RBC and Hb A_2_, but none in MCHC, RDW, Hct, Hb A and Hb F. The haematological parameters of nondeletional mutations are shown in Table 3.

The haematological parameters of compound heterozygous patients, including the CE analysis, are shown in Table 4. Furthermore, in the CE analysis, the Hb Constant Spring value among the compound heterozygous patients with Constant Spring genotypes had a mean value ± SD of 2.13 ± 1.06, while Hb H values of patients with --^SEA^/ α^Quong Sze^α, --^SEA^/α^CS^α and --^SEA^/-α^3.7^ were 6.4%, 2.4–12.2% and 5.1–12.2%, respectively. The Hb Barts mean values among compound heterozygous patients with --^SEA^/ α^Quong Sze^α, --^SEA^/α^CS^α and α^CS^α/ α^Adana^α were 29.2%, 0.9–1.8% and 0.8%, respectively.

On the other hand, in the compound heterozygous group, the majority of patients presented as Hb H phenotype, while some had fraction(s) of Hb H and/or Hb Barts. There were also three Hb Constant Spring variants identified in the compound heterozygous group with a mean ± SD value of 2.13 ± 1.06%.

## 4. Discussion

The prevalence of α-thalassaemia in this study was 48.9%. In a previous study, 22.5% of α-thalassaemia cases worldwide had been reported in Southeast Asian countries, and 17.5% of them were in Malaysia [3]. However, that prevalence varied among geographical areas and ethnicity. Malaysia is a multiracial country with a Malay majority, followed by Chinese, Indians and other small groups such as the Siamese. Kelantan state, which is where our institution is located, is next to south Thailand, a country that has a 20.1% prevalence of α-thalassaemia. Many people in northern Peninsular Malaysia are related to those in south Thailand due to intermarriage and migration throughout time [3,9].

The most common deletional mutation was the -α^3.7^ genotype, followed by αα^CS^, --^SEA^, -α^4.2^ and αα^Quong Sze^, and the least common was αα^Adana^. The findings were in accordance with a few local studies [10,11,12]. Similar distributions of mutations have been reported in neighbouring countries, including Thailand [13,14], Laos [15], Cambodia [16] and Vietnam [17,18]. Among nondeletional mutations, Hb Constant Spring was the most prevalent in this study, followed by Hb Quong Sze. These findings are similar to other local studies [11,19] and in Southeast Asia [5]. This study showed similar frequency patterns in which 10.3% of patients had Hb Constant Spring (*n* = 14), followed by Hb Quong Sze (*n* = 2, 1.5%).

Compound heterozygous α-thalassaemia patients had a different mutation in each α-globin chain. In this study, 6.6% of patients (*n* = 9) were diagnosed with compound heterozygous α-thalassaemia, which were --^SEA^/-α^3.7^ (*n* = 3) and --^SEA^/α^CS^α (*n* = 2), and one patient each with the following genotypes: -α^4.2^/α^CS^α, --^SEA^/α^Quong Sze^α, -α^3.7^/α^Adana^α and αα^CS^/α^Adana^α. Compound heterozygous --^SEA^/-α^3.7^ had only one unaffected α-globin gene leading to Hb H disease, which has been commonly reported worldwide. Similarly, Malaysia [20], Thailand [21] and Taiwan [22] had reported --^SEA^/-α^3.7^ as a common compound heterozygous deletional mutation in their population. This study showed that 2.2% of patients had --^SEA^/-^3.7^, and this was the most common compound heterozygous deletion detected in the studied population.

Single-gene deletion mutations, including -α^3.7^ and -α^4.2^, resulted in asymptomatic/mild clinical manifestations. Most carriers of a single globin gene deficiency had normal Hb due to a compensatory increase in the number of microcytic RBCs. Such individuals would not experience severe anaemia-related symptoms, such as fatigue or weakness [23]. If the diagnostic criteria solely depended on haematological parameters, then this would lead to misdiagnosis [9]. There were no significant differences in Hb levels between -α^4.2^ and -α^3.7^ patients. However, some parameters, including Hb, MCHC, RBC and Hct, were lower in the heterozygous 3.7 kbp α deletion patients compared with those who had the 4.2 kbp deletion, even though both had lower readings in these parameters. Previously, few studies reported mildly low to normal levels of MCV and MCH among similar genotypes [11,24,25]. In this study, there were no significant differences in the parameters between αα/αα and the heterozygous -α^3.7^ observed in the further statistical test, post hoc analysis. This is probably because the αα/αα group of suspected thalassaemia patients might have other conditions/diseases that affected haematological parameters.

The normal range of Hb A_2_ is between 2.2% and 3.2% [26]. Hb A_2_ levels in individuals with a single α-globin gene deletion are within the normal range. Consistent with that, in this study, the mean value of Hb A_2_ among the α-thalassaemia patients was 3.03%. However, a slight increase in Hb A_2_ levels was observed in two patients with normal Hb F. Hb A_2_ comprised two α-globin chains and two delta (δ)-globin chains (α2δ2), and its level was also a significant marker of β-thalassaemia. The defective β-globin gene could result in the absence or reduction of β-globin chain synthesis, leading to an excess of α-globin chains and subsequent formation of Hb A_2_ [27]. There were also other factors that could cause an increase in Hb A_2_ levels, including vitamin B12 deficiency, folate deficiency, antiretroviral therapy and hyperthyroidism [28]. In this study, the cause of elevated Hb A_2_ level was not further investigated, including in β-thalassaemia genotyping. No significant differences in haematological parameters and Hb quantification were also detected in single- and double-gene deletion patients. Under normal circumstances, the level of Hb F would decrease with increased gestational age. In adults, the normal Hb F level is <1%, and a decrease in Hb F has been reported with more defective α-globin genes [29]. However, no significant differences were identified among the single- and double-gene deletions in this study. Moreover, the level was within the normal range among the patients. A study suggested that the level of Hb F would increase with a greater number of β-globin genes affected in β-thalassaemia [28], whereas α-thalassaemia patients usually have normal Hb F levels [30].

The double-gene deletion showed a significantly lower level of MCV, MCH and MCHC compared with silent-trait groups [10,12]. A similar outcome was found in this study, in which --^SEA^ patients showed the lowest median values for MCV, MCH and MCHC compared with other deletional groups. In addition, --^SEA^ patients showed significantly lower levels of MCV and MCH than -α^3.7^ patients. The Hb level in a patient with the homozygous α^3.7^ genotype showed the lowest level among other deletional groups, followed by heterozygous --^SEA^ patients. The homozygous α^3.7^ had two deleted genes with a similar haematological parameter range as in the heterozygous α double-gene mutations. [11].

The Hb A_2_ level was within the normal range for all patients, and the lowest median for Hb A_2_ was observed in the heterozygous --^SEA^ mutation. Carriers of double-gene deletions usually present with a normal or slightly lower Hb A_2_ level [31]. RDW measures the variation in RBC size and plays an important role in differentiating between iron-deficiency anaemia (IDA) and thalassaemia traits. IDA patients commonly report higher RDW levels compared with thalassaemia [32]. The level of RDW in thalassaemia patients is usually normal or slightly increased, reflecting uniformity in RBC size and microcytes [26]. Thalassaemia patients in this study showed normal to slightly low RDW. However, the value of RDW increased with the increased number of defective genes in thalassaemia patients [11,29]. A similar finding has also been reported in which higher RDW levels were found in cases with more affected α-globin genes [12]. In contrast, a mild elevation in RDW has been observed in the α-thalassaemia trait compared with the normal genotype [11]. However, in this study, there were no significant differences among the different deletional mutations at the RDW level. In addition, the heterozygous --^SEA^ patient presented with the lowest median value of RDW compared with other deletional groups.

Nondeletional mutations show diverse haematological parameters depending on the mutated region and its interaction with α- and β-globin chains [33]. The mutation in the *HBA2* termination codon would result in elongated α-globin chains. This condition might lead to unstable RBC formation due to the weak binding of globin chains. Among the Hb Constant Spring patients, abnormal MCV has been reported [5]. Similarly, a study reported a decrease in levels of MCV, MCH and MCHC among heterozygous Hb Constant Spring patients [34]. In contrast, another study showed normal MCV, MCH and Hb A_2_ among the same patients [24]. Previous studies [24,34] have reported a wide range in various parameters; however, in the current study, Hb quantification showed normal Hb A, Hb A_2_ and Hb F levels in all subjects.

**Figure 3:**
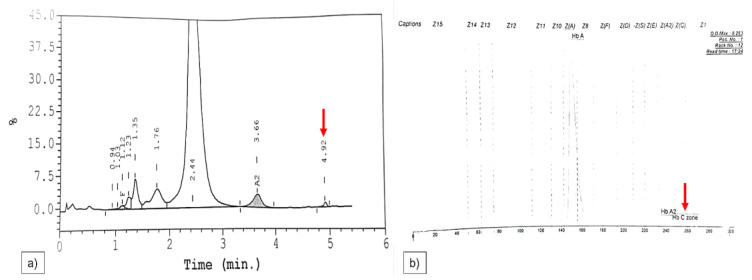
(**a**,**b**) A representative result of Hb Constant Spring by HPLC and CE analyses. The red arrow represents the Hb Constant Spring peak and zone in HPLC and CE respectively.

The Hb, MCV, MCH and MCHC of Hb Constant Spring patients showed a slight decrease, but it was not significant compared with other mutations. It was challenging to characterise the mutation solely depending on haematological parameters. In heterozygous patients, this variant made up 1 to 2% of total Hb. HPLC and CE could both detect this variant. However, individuals with a small amount of this Hb were often not detectable in HPLC, but this limitation could be resolved by using CE [35,36]. In HPLC and CE analyses, Hb Constant Spring would produce a peak in window C and zone 2, as shown in Figure 3.

Patients with Hb Quong Sze showed normal haematological parameters except for MCV and MCH, which were slightly lower compared with the normal range. Similar findings were also published; however, most of the studied population had mild to moderate anaemia [5]. Hb Quong Sze is undetectable in routine electrophoresis and, thus, requires molecular analysis for confirmation [37].

The point mutation in codon 59 in either HBA1 or HBA2 would lead to the production of an abnormal Hb known as Hb Adana. The MCV, MCH and MCHC levels in these patients were slightly lower, but they were not statistically different among the different mutation groups. A previous study reported that Hb Adana carriers had a normal to slight decrease in MCV levels or an increase in RBC levels [38]. Similarly, another study found the carriers had normal haematological parameters with mild anaemia [5]. On the other hand, this Hb variant is highly unstable, which cannot be detected in routine CE [38]. Thus, it could be missed if the diagnosis depended only on haematological parameters [5,38]. Therefore, multiplex ARMS-PCR and Sanger sequencing played an important role in ensuring an accurate diagnosis and the provision of genetic counselling.

Hb H disease reflects the presence of three defective α-globin genes (--/-α), and it can occur due to deletion or mutation. The disease is the symptomatic form of α-thalassaemia. The most prevalent type of Hb H disease is the deletional type, which is brought on by compound heterozygosity caused by a double-globin gene deletion on one allele and a single globin gene deletion on the other allele. Nondeletional Hb H disease is rare, and it has a more severe clinical presentation. The disease is usually caused by homozygosity for nondeletional alleles or compound heterozygous for point mutations in either α1- or α2-globin chains, with heterozygosity for double-gene deletion on one chromosome [39]. However, the clinical outcome and haematological parameters are widely variable, from very mild to severe phenotypes. The severity depends on the imbalanced production of α- and β-globin chains, which is determined by the underlying α-globin gene mutations [40]. There is a significant decrease in Hb, MCV, MCH and MCHC compared with cases with one or two defective α-globin genes [21].

All haematological parameters of deletional compound heterozygous (--^SEA^/-α^3.7^) showed low haematological parameters. The RDW was high in a patient with α^CS^α/α^Adana^α. The Hb Adana was a severe nondeletional mutation and commonly associated with Hb H-induced hydrops fetalis syndrome in compound heterozygous cases [41]. The RDW level has been reported to be elevated in Hb H disease patients [42]. The Hb H level was detected in zone 15 of the CE analysis, with a mean value of 8.9 ± 3.56. Comparatively, other compound heterozygous mutations in this study, including -α^4.2^/α^CS^α, --^SEA^/α^CS^α, --^SEA^/α^Quong Sze^α, -α^3.7^/α^Adana^α, and α^CS^α/α^Adana^α, showed the presence of Hb Barts. A decrease in the production of α-globin chains due to the mutation on HBA1 and HBA2 leads to excess gamma (γ) chain production, which joins together to form an unstable tetramer called Hb Barts [43]. This causes the formation of Hb Barts, which is usually observed in patients with α-thalassaemia major.

The involvement of nondeletional mutations in α-globin genes can cause severe and significant clinical outcomes that might require regular blood transfusion [44]. The nondeletional mutations result in unstable Hb production, which leads to the formation of damaged RBCs. However, the most unstable Hb is produced when nondeletional mutations interact with deletional ones [44]. The haematological parameters were lower in all patients with Hb H disease in this study. Hb, MCV, MCH, Hct and Hb F were found to be lower in patients with deletional compound heterozygous (--^SEA^/-α^3.7^) compared with other mutations. A contradictory finding was reported, in which cases of compound nondeletional mutations showed lower values for most of the haematological parameters [21]. Therefore, molecular analysis is necessary to understand the pathology and, more importantly, provide correct genetic counselling and prenatal diagnosis in high-risk families. Despite the results obtained in this study, this research has several limitations, such as investigation into the lack of iron to rule out coinheritance IDA, and the selection of patients based on haematological parameters, resulting in the inability to identify α-thalassaemia in asymptomatic patients.

## 5. Conclusions

This study found a 48.9% prevalence of α-thalassaemia in the patient cohort. The Hb, MCV, MCH, MCHC, RBC, RDW, Hct, Hb A, Hb A_2_ and Hb F levels in patients, including those with the same genotypes, revealed variations that might be due to environmental and genetic modifiers. Thus, haematological parameters alone would not be specific enough to describe each α-thalassaemia mutation. A combination of molecular techniques, including multiplex gap-PCR, multiplex ARMS-PCR, MLPA and Sanger sequencing, would be helpful for the accurate and precise detection of α-globin chain mutations.

## Figures and Tables

**Table 1:** Types of α-globin genotypes identified.

Type of α-Globin Genotype	Frequency	Percentage (%)
**Deletional**		
-α^3.7^/αα	21	15.4
-α^4.2^/αα	5	3.7
--^SEA^/αα	10	7.4
-α^3.7^/-α^3.7^	1	0.7
	37	57.8
**Nondeletional**		
α^CS^α/αα	14	10.3
α^Adana^α/αα	1	0.7
α^Quong Sze^α/αα	2	1.5
α^CS^α/α^CS^α	1	0.7
	18	28.1
**Compound heterozygous**		
-α^4.2^/α^CS^α	1	0.7
--^SEA^/α^CS^α	2	1.5
--^SEA^/α^Quong Sze^α	1	0.7
-α^3.7^/α^Adana^α	1	0.7
--^SEA^/-α^3.7^	3	2.2
α^CS^α/α^Adana^α	1	0.7
	9	14.1

**Table 2:** Haematological parameters among deletional mutations of α-thalassaemia patient.

Parameters (Median, Interquartile Range)	αα/αα *n* = 67	-α^3.7^/αα *n* = 21	-α^3.7^/-α^3.7^ *n* = 1	-α^4.2^/αα *n* = 5	--^SEA^/αα *n* = 10	*p*-Value
Hb (g/dL)	11.4 (8.8–12.7)	12.0 (10.0–13.7)	8.1	13.6 (13.1–16.85)	11.55 (10.78–12.53)	0.022 *
MCV (fL)	71.1 (65.4–74.8)	76.8 (68.85–81.75)	77.1	70.9 (70.85–79.65)	66.35 (62.68–70.68)	0.009 *
MCH (pg)	22.7 (19.1–24.5)	25.5 (20.75–26.50)	22.9	24.1 (23.3–26.55)	20.5 (19.78–22.3)	0.017 *
MCHC (g/dL)	31.7 (29.6–32.9)	31.70 (30.90–33.10)	29.8	34.0 (31.8–34.45)	31.4 (31.25–31.85)	0.173 ^ns^
RBC (10^12^/L)	4.97 (4.43–5.48)	4.92 (4.59–5.45)	3.53	5.5 (4.72–6.45)	5.83 (5.25–6.22)	0.038 *
RDW (fL)	39.9 (35.4–44.7)	40.8 (37.3–43.1)	44.9	40.0 (39.8–50.85)	34.8 (33.1–38.75)	0.060 ^ns^
Hct (%)	35.6 (30.6–39.8)	36.3 (33.15–42.4)	27.2	40.0 (39.8–50.85)	37.15 (33.85–40.03)	0.058 *
Hb A (%)	96.4 (72.3–97.1)	96.8 (96.1–96.95)	96.1	96.4 (95.45–96.55)	96.6 (96.0–97.13)	0.747 ^ns^
Hb A_2_ (%)	2.9 (2.6–26.4)	2.8 (2.65–3.1)	3.2	3.2 (2.95–3.2)	2.45 (2.35–2.75)	0.048 ^ns^
Hb F (%)	0.6 (0.3–0.9)	0.4 (0.3–0.75)	0.7	0.4 (0.4–1.45)	0.6 (0.38–1.25)	0.388 ^ns^

Note: ns indicates nonsignificant *p*-value. * Indicates *p* < 0.05 and statistically significant. Kruskal–Wallis test was performed.

**Table 3:** Haematological parameters among nondeletional mutations of α-thalassaemia patients.

Parameters (Median, Interquartile Range)	αα/αα *n* = 64	α^CS^α/αα *n* = 14	α^CS^α/α^CS^α *n* = 1	α^Adana^α/αα *n* = 1	α^Quong Sze^α/αα *n* = 2
Hb (g/dL)	11.3 (8.73–12.68)	12.4 (11.55–13.55)	10	15.3	13.65 (12.5–14.8)
MCV (fL)	71.25 (65.1–74.95)	79.55 (70.48–80.55)	70.9	79.8	70.3 (64.7–75.9)
MCH (pg)	22.7 (19.1–24.48)	25.15 (23.35–26.0)	21.1	23.8	24.95 (22.9–27.0)
MCHC (g/dL)	31.75 (29.68–32.9)	25.15 (23.35–26.0)	29.8	29.8	35.5 (35.4–35.6)
RBC (10^12^/L)	4.96 (4.44–5.46)	5.14 (4.78–5.50)	4.74	6.44	9.2 (5.46–12.94)
RDW (fL)	39.8 (35.5–44.6)	37.84 (35.95–40.43)	40.7	45.2	38.8 (36.6–41.0)
Hct (%)	35.6 (30.3–39.68)	38.25 (36.1–42.93)	33.6	51.4	38.15 (35.3–41.0)
Hb A (%)	96.4 (72.33–97.08)	96.7 (96.3–96.93)	94.4	96.6	96.55 (96.3–96.8)
HbA_2_ (%)	2.9 (2.45–26.25)	2.7 (2.48–2.9)	2.1	3	2.7 (2.6–2.8)
Hb F (%)	0.6 (0.3–0.9)	0.55 (0.48–0.9)	2.1	0.4	0.75 (0.4–1.1)

**Table 4:** Haematological parameters between compound heterozygous α-thalassaemia patients.

Genotype	--^SEA^/-α^3.7^ *n* = 3	^--SEA^/α^Quong Sze^α *n* = 1	^--SEA^/α^CS^α *n* = 2	-α^4.2^/α^CS^α *n* = 1	-α^3.7^/α^Adana^α *n* = 1	α^CS^α/α^Adana^α *n* = 1
Parameters/ Phenotype	Deletional Hb H		Nondeletional Hb H			Hb H phenotype
Hb (g/dL)	8.2 (6.5–10.3)	6.7	5.8–9.5	10.9	9.2	7.0
MCV (fL)	60.6 (57.6–67.7)	71.1	75.4–78.7	64	65.9	90.1
MCH (pg)	18.5 (16.9–21)	18.5	20.1–24.1	19.7	21.3	24.7
MCHC (g/dL)	30.5 (29.3–31)	26.5	26.6–30.6	30.8	32.4	27.5
RBC (10^12^/L)	4.44 (3.1–6.11)	3.56	2.89–3.94	5.53	4.31	2.83
RDW (fL)	44.1 (40.9–45.2)	59	40.1–57.1	33.2	36.4	82.0
HCT (%)	26.9 (21–35.2)	25.3	21.8–31	35.4	28.4	25.5
Hb A (%)	98.1 (98–98.4)	52.7	81.7–85	96.9	96.7	80.0
Hb A_2_ (%)	1.5 (1.4–1.8)	1.1	0.7–2.9	2.4	2.5	2.5
Hb F (%)	0.2 (0.2–0.4)	14.5	0.5–1	1.1	0.8	3.6
Hb H (%)	9.4 (5.1–12.2)	6.4	2.4–12.2	-	-	-
Hb Barts	-	29.2%	0.9–1.85	-	-	0.8

The parameters for --^SEA^/-α^3.7^ are shown as median and interquartile range.

## Data Availability

The data presented in this study are available on request from the corresponding author.

## Supplementary Materials

The following supporting information can be downloaded at: https://www.mdpi.com/article/10.3390/diagnostics13050894/s1, Table S1: List of primers and concentrations used in multiplex gap-PCR, Table S2: List of primers and concentrations used in multiplex ARMS-PCR.
