# Supplementary material for: Gene Mutation Spectrum among Alpha-Thalassaemia Patients in Northeast Peninsular Malaysia
Source: Diagnostics (Basel). 2023 Feb 27;13(5):894. doi: 10.3390/diagnostics13050894 (PMC10000533; doi:10.3390/diagnostics13050894)
Supplement: Supplementary file 1 [file diagnostics-13-00894-s001.zip › diagnostics-2159647-supplementary.pdf]

Table S1. List of primers and concentrations used in multiplex gap-PCR.

Primers	Final Concentration
α2/3.7 F	0.15 μM
3.7/20.5 R	0.15 μM
SEA F	0.15 μM
SEA R	0.15 μM
α2 R	0.1 μM
20.5 F	0.1 μM
MED F	0.2 μM
MED R	0.2 μM
THAI F	0.2 μM
THAI R	0.2 μM
4.2 F	0.2 μM
4.2 R	0.2 μM
FIL F	0.2 μM
FIL R	0.2 μM
LIS * F	0.25 μM
LIS * R	0.25 μM

* Internal control, F (Forward), R (Reverse).

Table S2. List of primers and concentrations used in multiplex ARMS-PCR.

Primers	Final Concentration
Normal * F	0.2 μM
Normal * R	0.2 μM
Initiation codon, codon 30, 35, 59, 125 and 142 R	0.2 μM
Initiation codon F	0.2 μM
Codon 30 F	0.2 μM
Codon 35 F	0.2 μM
Codon 59 F	0.2 μM
Codon 125 F	0.2 μM
Codon 142 F	0.2 μM

* Internal control, F (Forward), R (Reverse).
